# Supplementary material for: Lipid droplets are a metabolic vulnerability in melanoma
Source: Nat Commun. 2023 Jun 2;14:3192. doi: 10.1038/s41467-023-38831-9 (PMC10238408; doi:10.1038/s41467-023-38831-9)
Supplement: Supplementary file 1 — Supplementary Information [file 41467_2023_38831_MOESM1_ESM.pdf]

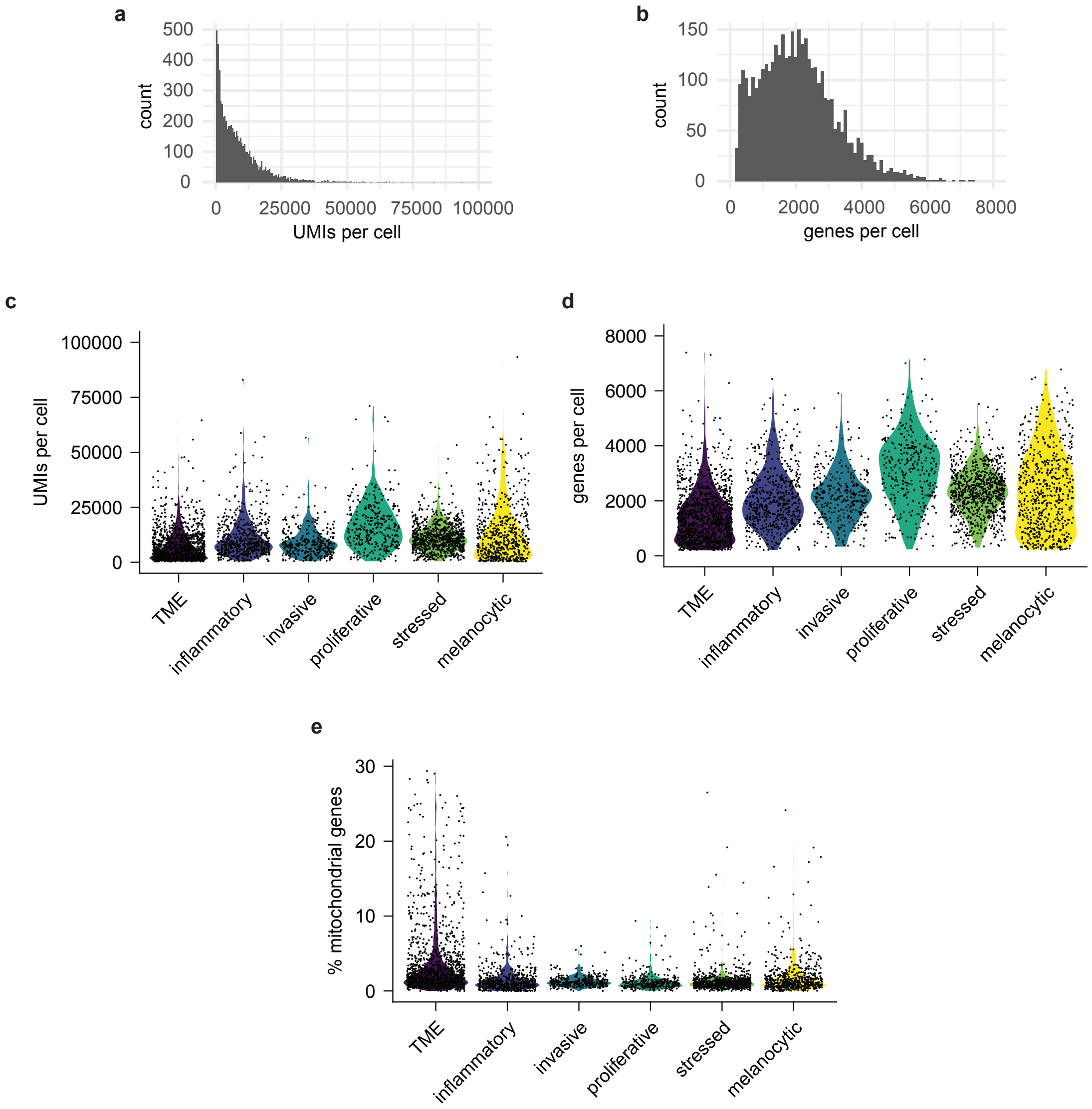
a
500
400
300
200
100
0
count
0
25000
50000
75000
100000
UMIs per cell
b
150
100
50
0
count
0
2000
4000
6000
8000
genes per cell
c
100000
75000
50000
25000
0
UMIs per cell
TME
inflammatory
invasive
proliferative
stressed
melanocytic
d
8000
6000
4000
2000
0
genes per cell
TME
inflammatory
invasive
proliferative
stressed
melanocytic
e
30
20
10
0
% mitochondrial genes
TME
inflammatory
invasive
proliferative
stressed
melanocytic

Supplementary Fig. 1: scRNA-seq data statistics.
a-b, Histograms of unique molecular identifiers (UMIs): a, and number of unique genes per cell: b, in the total data set of 3968 zebrafish melanoma and TME cells. c-d, Violin plot of UMIs per cell: c, unique genes per cell: d, and percent mitochondrial genes per cell: e, in each cluster assignment.

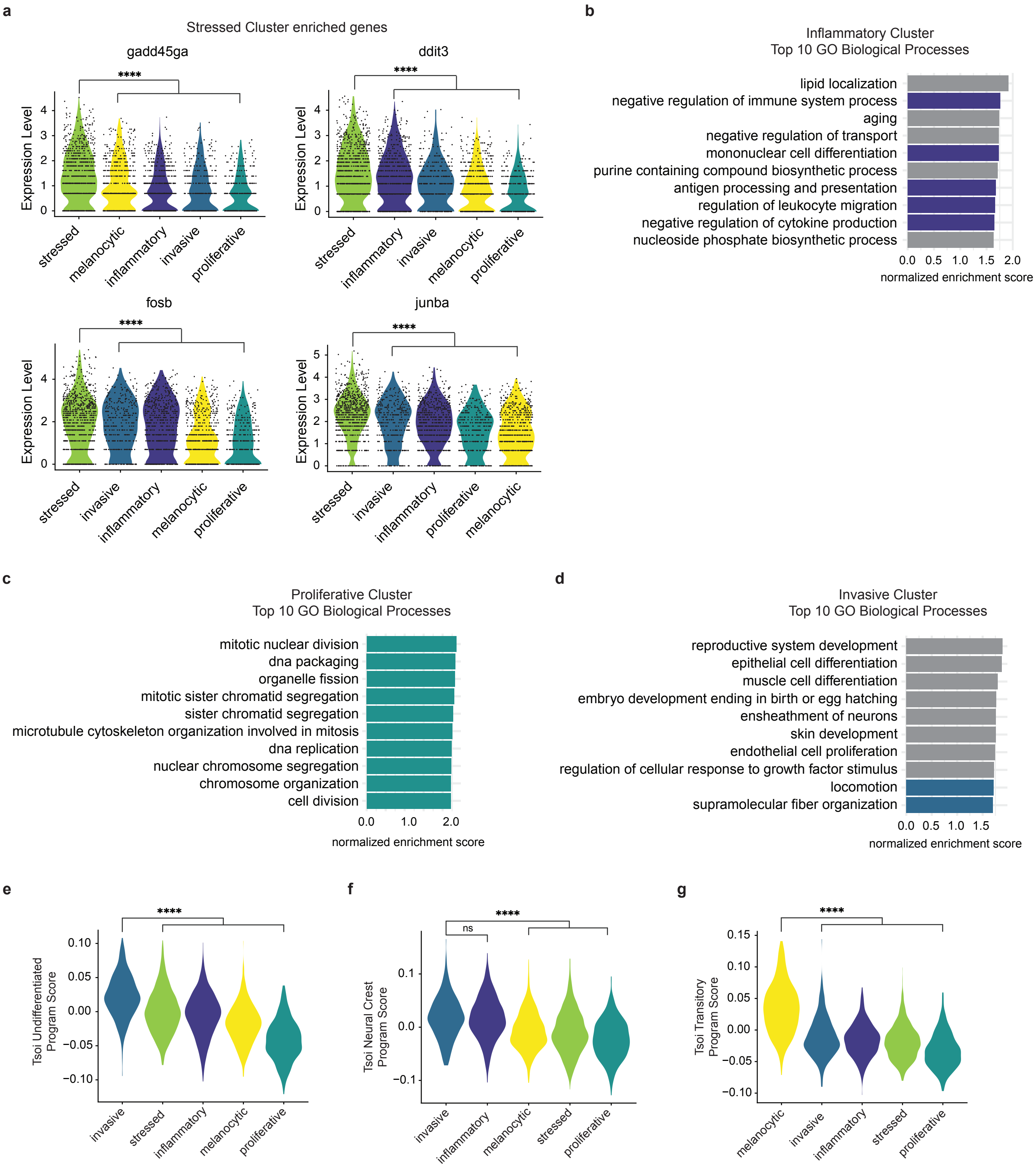
a
Stressed Cluster enriched genes
gadd45ga
ddit3
fosb
junba
****
Expression Level
stressed
melanocytic
inflammatory
invasive
proliferative
b
Inflammatory Cluster
Top 10 GO Biological Processes
lipid localization
negative regulation of immune system process
aging
negative regulation of transport
mononuclear cell differentiation
purine containing compound biosynthetic process
antigen processing and presentation
regulation of leukocyte migration
negative regulation of cytokine production
nucleoside phosphate biosynthetic process
normalized enrichment score
c
Proliferative Cluster
Top 10 GO Biological Processes
mitotic nuclear division
dna packaging
organelle fission
mitotic sister chromatid segregation
sister chromatid segregation
microtubule cytoskeleton organization involved in mitosis
dna replication
nuclear chromosome segregation
chromosome organization
cell division
normalized enrichment score
d
Invasive Cluster
Top 10 GO Biological Processes
reproductive system development
epithelial cell differentiation
muscle cell differentiation
embryo development ending in birth or egg hatching
ensheathment of neurons
skin development
endothelial cell proliferation
regulation of cellular response to growth factor stimulus
locomotion
supramolecular fiber organization
normalized enrichment score
e
Tsoi Undifferentiated Program Score
f
ns
Tsoi Neural Crest Program Score
g
Tsoi Transitory Program Score

Supplementary Fig. 2: Expression of differentially expressed genes and pathways in stressed, inflammatory, proliferative, and invasive melanoma clusters.

a, Violin plot of genes associated with cell stress that are differentially upregulated in the stressed cluster. Statistics via two-sided Wilcoxon rank-sum test[22]. **** p<0.0001. b-d, Top 10 enriched GO Biological Processes in inflammatory: b, proliferative: c, and invasive: d, clusters. Pathways are color coded based on relevance to cluster annotation in Fig. 1b. e-g, Tsoi Undifferentiated, Neural Crest Program, and Transitory4 module scores for cells in each melanoma cell state. Adjusted p-values calculated using two-sided Wilcoxon rank-sum test with Holm correction. **** p<0.0001.

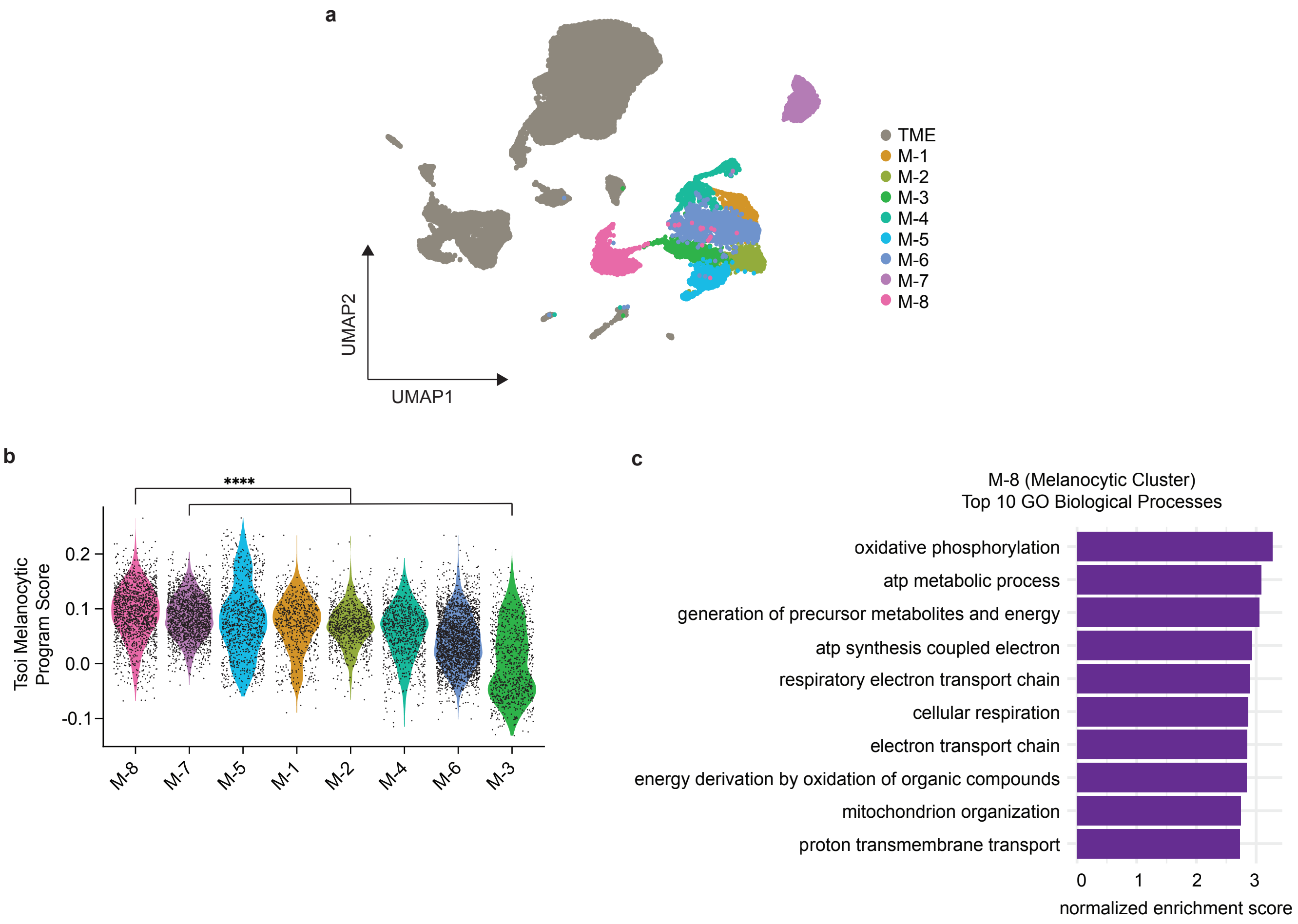
a
TME
M-1
M-2
M-3
M-4
M-5
M-6
M-7
M-8
UMAP2
UMAP1
b
****
Tsoi Melanocytic Program Score
0.2
0.1
0.0
-0.1
M-8
M-7
M-5
M-1
M-2
M-4
M-6
M-3
c
M-8 (Melanocytic Cluster)
Top 10 GO Biological Processes
oxidative phosphorylation
atp metabolic process
generation of precursor metabolites and energy
atp synthesis coupled electron
respiratory electron transport chain
cellular respiration
electron transport chain
energy derivation by oxidation of organic compounds
mitochondrion organization
proton transmembrane transport
0
1
2
3
normalized enrichment score

Supplementary Fig. 3: Melanocytic cell state enriches for oxidative metabolic pathways in human melanoma brain and leptomeningeal metastases.

a, UMAP projection of human melanoma brain and leptomeningeal metastases from Smalley et al.[24]. Unsupervised clustering[22] identified eight different melanoma clusters and TME cells as indicated. b, Melanoma cluster 8 (M-8) most enriches for Tsoi Melanocytic Program[4] module score. Adjusted p-values calculated using two-sided Wilcoxon rank-sum test with Holm correction. **** $p<0.0001$. c, Top 10 enriched GO Biological Processes by multi-level Monte Carlo with Benjamini-Hochberg adjusted p-values<0.01 in M-8 (Melanocytic Cluster) showing oxidative metabolic pathways.

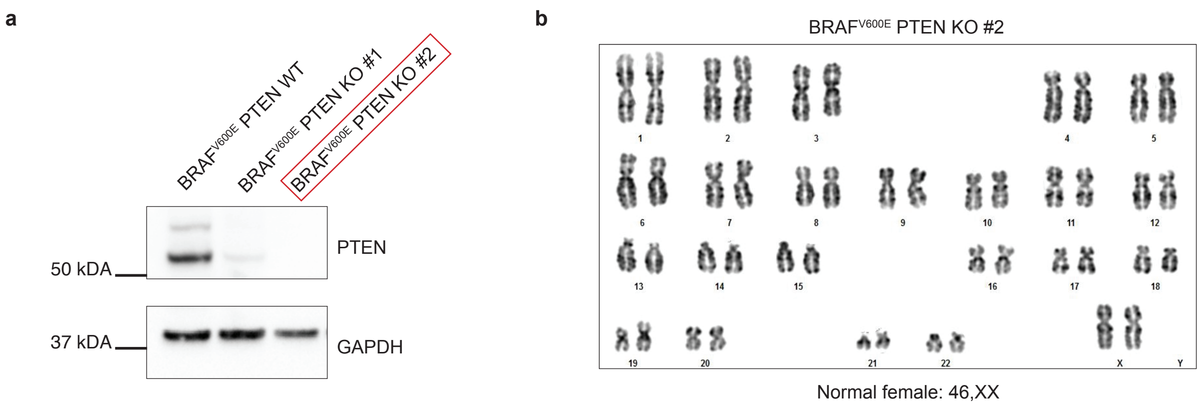
a
BRAFV600E PTEN WT
BRAFV600E PTEN KO #1
BRAFV600E PTEN KO #2
PTEN
50 kDA
37 kDA
GAPDH
b
BRAFV600E PTEN KO #2
1
2
3
4
5
6
7
8
9
10
11
12
13
14
15
16
17
18
19
20
21
22
X
Y
Normal female: 46,XX

Supplementary Fig. 4: Human pluripotent stem cells (hPSC) PTEN knockout line.
a, The doxycycline (dox)-inducible $BRAF^{V600E}$ hPSC line[13] was genetically engineered to be knockout (KO) for PTEN. Western blotting validates the full loss of PTEN in the KO line #2. b, Karyotypic analysis of the dox-inducible $BRAF^{V600E}$ PTEN KO hPSC line used in this study shows no major chromosomal abnormalities.

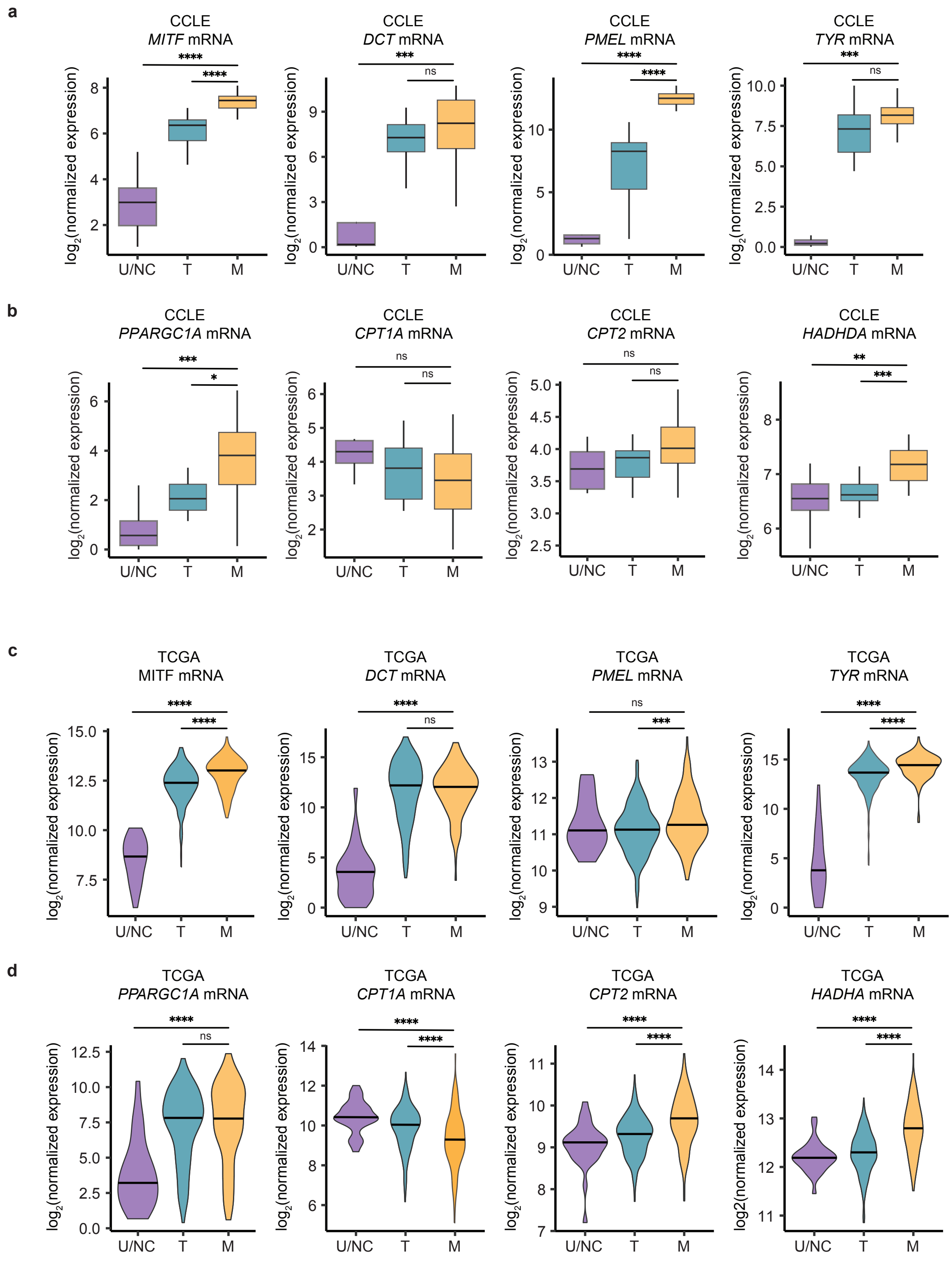
a
CCLE MITF mRNA
CCLE DCT mRNA
CCLE PMEL mRNA
CCLE TYR mRNA
b
CCLE PPARGC1A mRNA
CCLE CPT1A mRNA
CCLE CPT2 mRNA
CCLE HADHDA mRNA
c
TCGA MITF mRNA
TCGA DCT mRNA
TCGA PMEL mRNA
TCGA TYR mRNA
d
TCGA PPARGC1A mRNA
TCGA CPT1A mRNA
TCGA CPT2 mRNA
TCGA HADHA mRNA
log2(normalized expression)
U/NC
T
M
ns

Supplementary Fig. 5: Melanocytic and fatty acid oxidation gene expression.
a-b, CCLE $\log_2$ normalized mRNA expression for select: a, melanocytic and b, fatty acid oxidation genes. Data represented as Tukey's boxplot center = median, box bounds = 25th and 75th percentile, whiskers = min and max for undifferentiated/neural crest (U/N) n = 8, transitory (T) n = 12, melanocytic (M) n = 18. * $p<0.05$, ** $p<0.01$, *** $p<0.001$, **** $p<0.0001$. c-d, TCGA $\log_2$ normalized mRNA expression for select: c, melanocytic and d, fatty acid oxidation genes. Data represented as violin plot with mean for (U/N) n = 29, (T) n = 291, and (M) n = 123. Statistics via two-sided Wilcoxon rank sum test with Holm correction. *** $p<0.001$, **** $p<0.0001$.

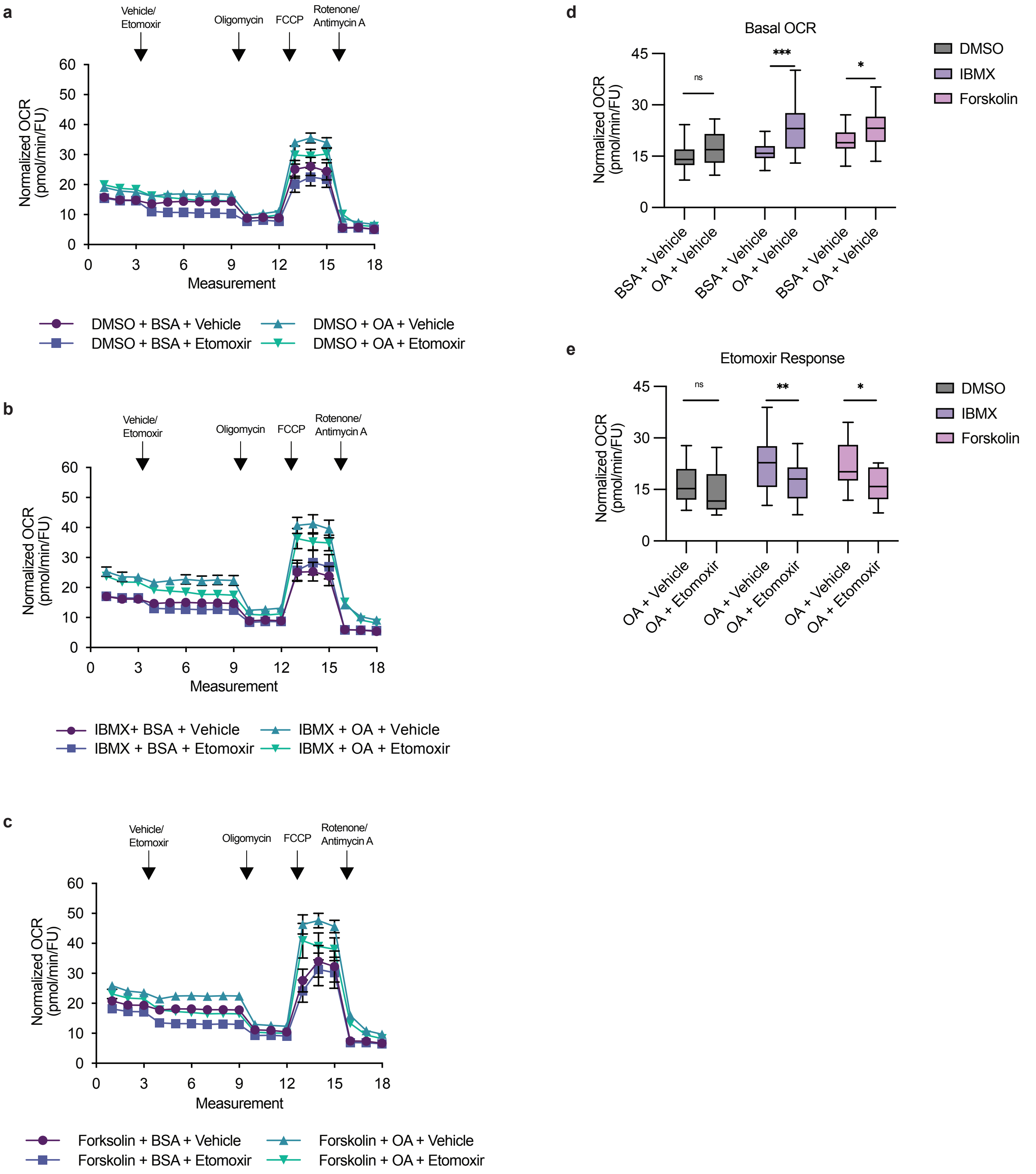
a
Vehicle/
Etomoxir
Oligomycin
FCCP
Rotenone/
Antimycin A
Normalized OCR
(pmol/min/FU)
Measurement
DMSO + BSA + Vehicle
DMSO + BSA + Etomoxir
DMSO + OA + Vehicle
DMSO + OA + Etomoxir
b
Vehicle/
Etomoxir
Oligomycin
FCCP
Rotenone/
Antimycin A
Normalized OCR
(pmol/min/FU)
Measurement
IBMX+ BSA + Vehicle
IBMX + BSA + Etomoxir
IBMX + OA + Vehicle
IBMX + OA + Etomoxir
c
Vehicle/
Etomoxir
Oligomycin
FCCP
Rotenone/
Antimycin A
Normalized OCR
(pmol/min/FU)
Measurement
Forksolin + BSA + Vehicle
Forskolin + BSA + Etomoxir
Forskolin + OA + Vehicle
Forskolin + OA + Etomoxir
d
Basal OCR
Normalized OCR
(pmol/min/FU)
ns
***
*
BSA + Vehicle
OA + Vehicle
DMSO
IBMX
Forskolin
e
Etomoxir Response
Normalized OCR
(pmol/min/FU)
ns
**
*
OA + Vehicle
OA + Etomoxir
DMSO
IBMX
Forskolin

Supplementary Fig. 6: Melanocytic cells utilize fatty acids for beta-oxidation.
a-c, Representative normalized OCR measurements for FAO stress test in DMSO (a), IBMX (b) or Forskolin (c) treated human A375 cells (mean ± SEM, n = 3 biologically independent experiments). Measurements are plotted separately for ease of visualization. d, Basal OCR from measurement 3 reported in normalized OCR values (Tukey's boxplot center = median, box bounds = 25th and 75th percentile, whiskers = min and max, n = 3 biologically independent experiments). * p=0.01, *** p<0.001. e, Etomoxir response from measurement 9 reported in normalized OCR values (Tukey's boxplot center = median, box bounds = 25th and 75th percentile, whiskers = min and max, n = 3 biologically independent experiments). Statistics via two-tailed t-test with Holm-Sidak correction. * p=0.04, ** p=0.004.

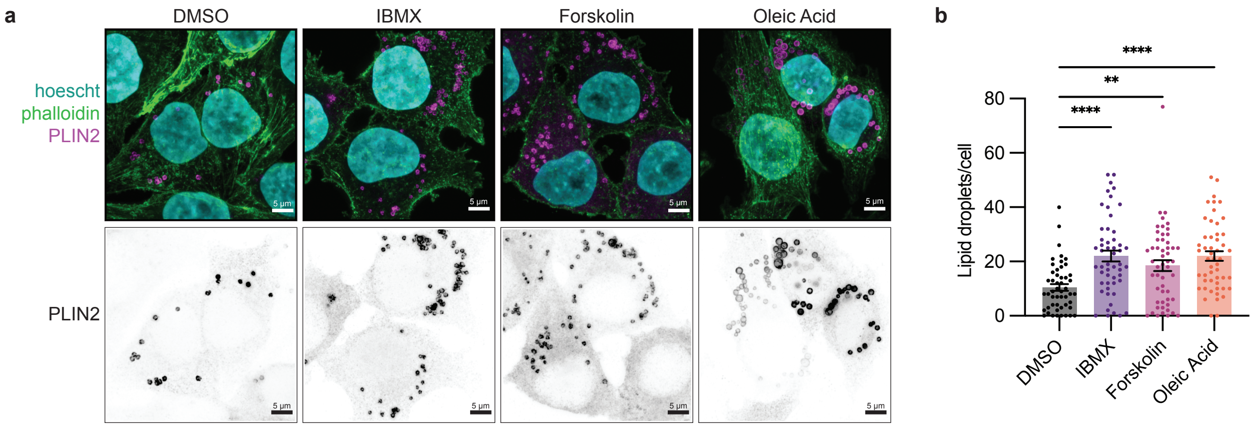
a
DMSO
IBMX
Forskolin
Oleic Acid
hoescht
phalloidin
PLIN2
PLIN2
5 µm
b
****
**
****
80
60
40
20
0
Lipid droplets/cell
DMSO
IBMX
Forskolin
Oleic Acid

Supplementary Fig. 7: A375 cells increase lipid droplet production upon acquisition of melanocytic cell state.

a-b, (a) Representative fluorescent images of PLIN2+ lipid droplets in drug treated human A375 cells and (b) corresponding quantification of PLIN2+ lipid droplets per cell (mean ± SEM, DMSO n = 50 biologically independent replicates, IBMX n = 52 biologically independent replicates, Forskolin n = 52 biologically independent replicates, OA n = 51 biologically independent replicates over n = 3 biologically independent experiments). Statistics via Kruskal Wallis with Dunn's multiple comparisons test. ** p=0.004, **** p<0.0001.

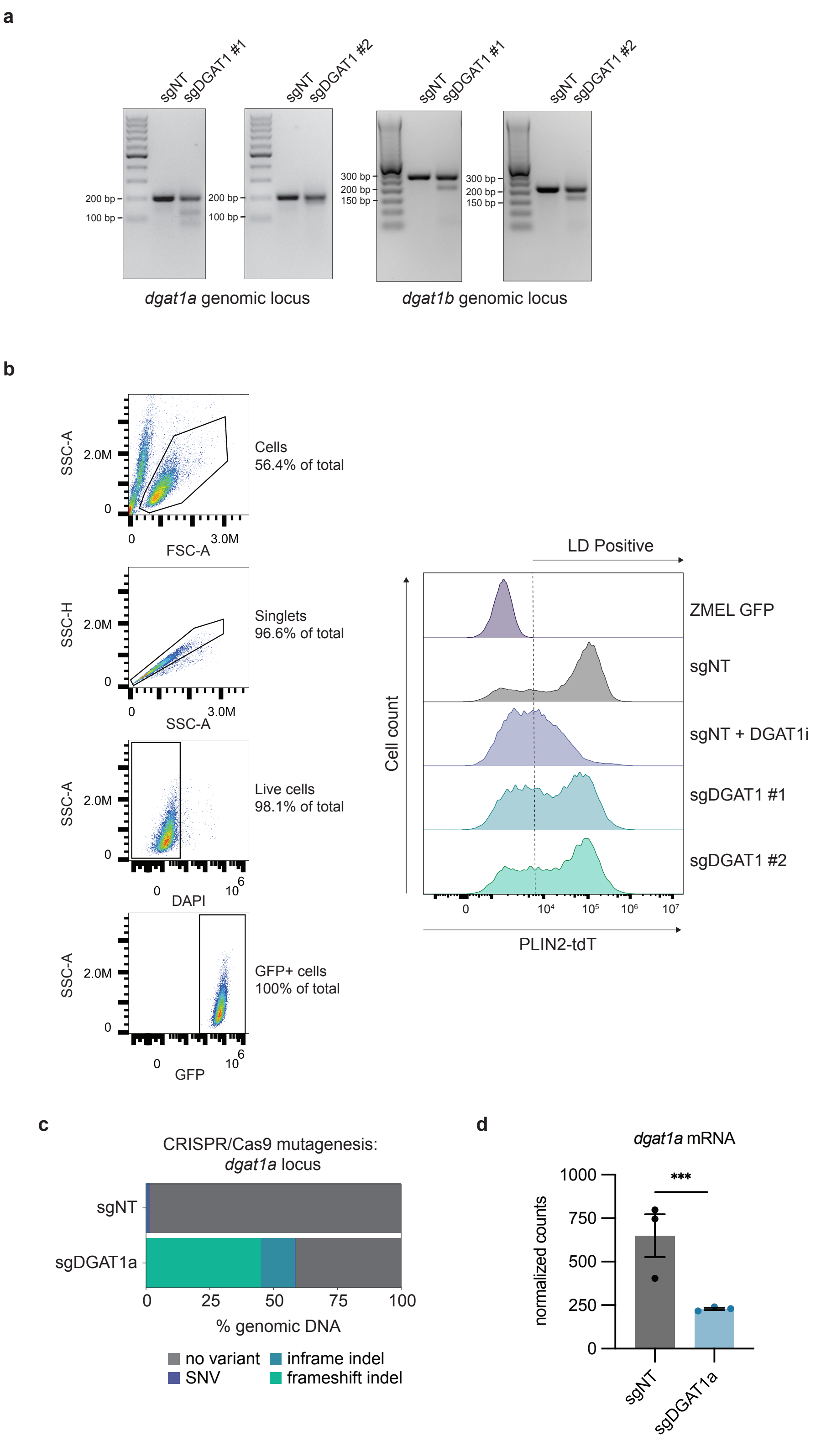
a
sgNT
sgDGAT1 #1
sgNT
sgDGAT1 #2
sgNT
sgDGAT1 #1
sgNT
sgDGAT1 #2
200 bp
100 bp
200 bp
100 bp
300 bp
200 bp
150 bp
300 bp
200 bp
150 bp
dgat1a genomic locus
dgat1b genomic locus
b
SSC-A
2.0M
0
0
3.0M
FSC-A
Cells
56.4% of total
SSC-H
2.0M
0
0
3.0M
SSC-A
Singlets
96.6% of total
SSC-A
2.0M
0
0
10^6
DAPI
Live cells
98.1% of total
SSC-A
2.0M
0
0
10^6
GFP
GFP+ cells
100% of total
LD Positive
Cell count
ZMEL GFP
sgNT
sgNT + DGAT1i
sgDGAT1 #1
sgDGAT1 #2
0
10⁴
10⁵
10⁶
10⁷
PLIN2-tdT
c
CRISPR/Cas9 mutagenesis:
dgat1a locus
sgNT
sgDGAT1a
0
25
50
75
100
% genomic DNA
no variant
inframe indel
SNV
frameshift indel
d
dgat1a mRNA
1000
750
500
250
0
normalized counts
***
sgNT
sgDGAT1a

Supplementary Fig. 8: Validation of *dgat1a* and DGAT1 perturbation in zebrafish and human melanoma cells.

a, Surveyor nuclease assay for sgDGAT1 guide sets demonstrating indels introduced at the *dgat1a* and *dgat1b* genomic loci. Blots run for n = 3 biologically independent experiments. b, Gating strategy and histogram of PLIN2-tdTomato expression in ZMEL-LD cells with DGAT1 perturbation. ZMEL GFP cells devoid of tdTomato expression are negative control for PLIN2-tdTomato. c, Bar plot of CRISPR/Cas9 mutagenesis in *dgat1a* locus of sorted zebrafish melanomas as indicated from Fig. 6a. d, Normalized counts for *dgat1a* mRNA in sorted zebrafish melanomas as indicated from Fig. 6a (mean ± SEM, n = 3 zebrafish per genotype). Statistics via Wald test. *** $p<0.001$.

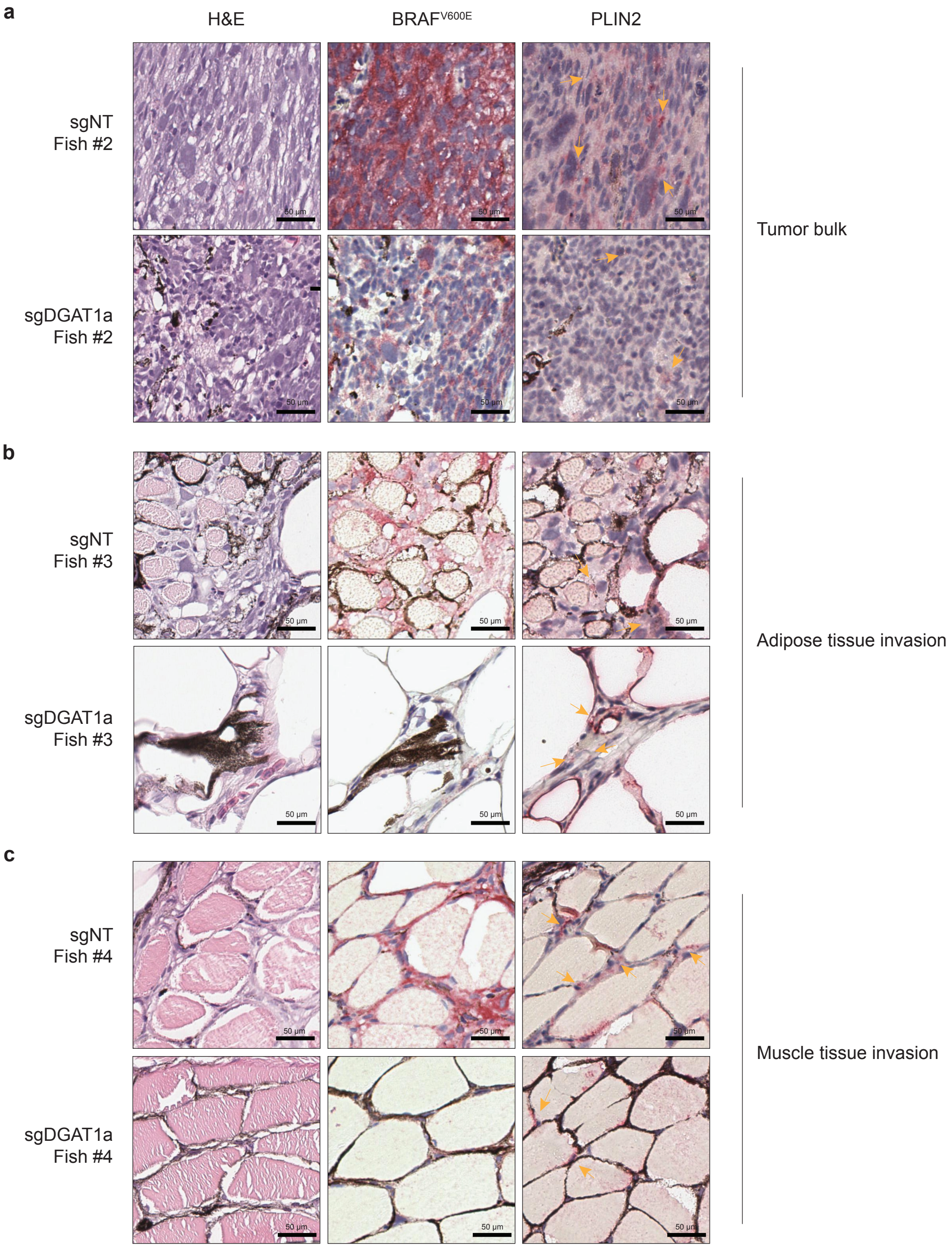
a
H&E
BRAFV600E
PLIN2
sgNT
Fish #2
sgDGAT1a
Fish #2
Tumor bulk
b
sgNT
Fish #3
sgDGAT1a
Fish #3
Adipose tissue invasion
c
sgNT
Fish #4
sgDGAT1a
Fish #4
Muscle tissue invasion
50 μm

Supplementary Fig. 9: Decreased PLIN2 staining in *dgat1a* knockout tumors.
a-c, Representative histological images from individual TEAZ generated tumors (n = 4 fish per genotype) in matched areas stained with H&E, $BRAF^{V600E}$, and PLIN2. Yellow arrows indicate punctate PLIN2 staining in the: (a) tumor bulk, (b) adipose tissue invasion, and (c) muscle tissue invasion.

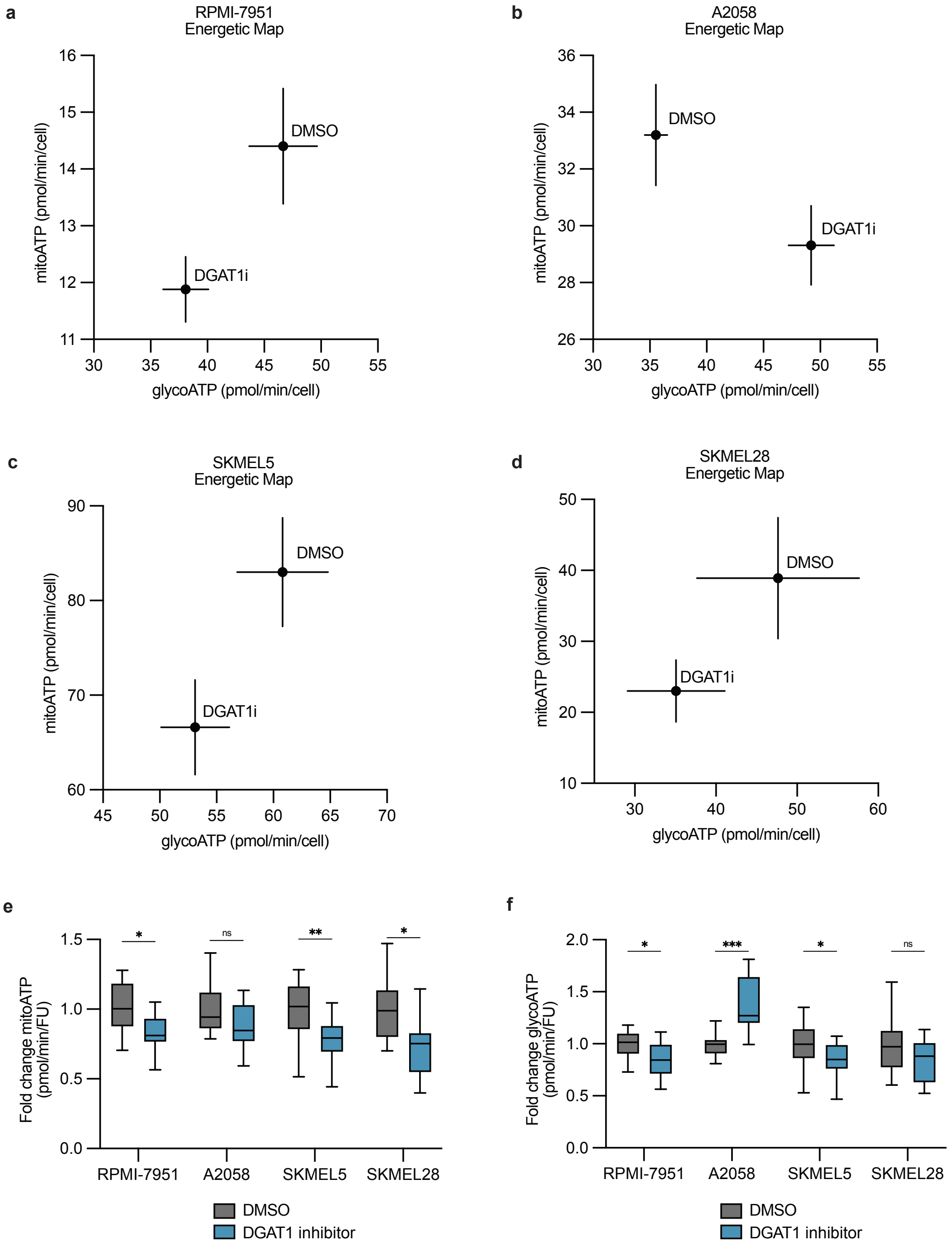
a
RPMI-7951
Energetic Map
mitoATP (pmol/min/cell)
glycoATP (pmol/min/cell)
DMSO
DGAT1i
b
A2058
Energetic Map
mitoATP (pmol/min/cell)
glycoATP (pmol/min/cell)
DMSO
DGAT1i
c
SKMEL5
Energetic Map
mitoATP (pmol/min/cell)
glycoATP (pmol/min/cell)
DMSO
DGAT1i
d
SKMEL28
Energetic Map
mitoATP (pmol/min/cell)
glycoATP (pmol/min/cell)
DMSO
DGAT1i
e
Fold change mitoATP (pmol/min/FU)
RPMI-7951
A2058
SKMEL5
SKMEL28
*
ns
**
*
DMSO
DGAT1 inhibitor
f
Fold change glycoATP (pmol/min/FU)
RPMI-7951
A2058
SKMEL5
SKMEL28
*
***
*
ns
DMSO
DGAT1 inhibitor

Supplementary Fig. 10: DGAT1 inhibition disrupts metabolic homeostasis.
a-d, Seahorse ATP Rate Energetic Maps for (a) RPMI-7951, (b) A2058, (c) SKMEL5, and (d) SKMEL28 cells (mean ± SEM, n = 3 biologically independent experiments). e-f, Fold change of (e) mitoATP and (f) glycoATP treated with DMSO or 40 μM DGAT1 inhibitor (Tukey's boxplot center = median, box bounds = 25th and 75th percentile, whiskers = min and max, n = 3 biologically independent experiments). Statistics via two-sided Mann-Whitney test with Holm-Sidak correction. * $p<0.05$, ** $p<0.01$, *** $p<0.001$.
