# Supplementary material for: Lipid droplets are a metabolic vulnerability in melanoma
Source: Nat Commun. 2023 Jun 2;14:3192. doi: 10.1038/s41467-023-38831-9 (PMC10238408; doi:10.1038/s41467-023-38831-9)
Supplement: Supplementary file 3 — Description of Additional Supplementary Files [file 41467_2023_38831_MOESM3_ESM.pdf]

File name: Supplementary Data 1

Description: Single-cell RNA sequencing of zebrafish melanomas generated by transgene electroporation. Tabs represent the 5 main melanoma cell clusters that correspond to similar clusters seen in human melanoma.

File name: Supplementary Data 2

Description: Bulk RNA sequencing of zebrafish melanomas with or without DGAT1 CRISPR. Melanoma cells were sorted using the tdTomato transgene.
