# Supplementary material for: Lipid droplets are a metabolic vulnerability in melanoma
Source: Nat Commun. 2023 Jun 2;14:3192. doi: 10.1038/s41467-023-38831-9 (PMC10238408; doi:10.1038/s41467-023-38831-9)
Supplement: Supplementary file 6 — Reporting Summary [file 41467_2023_38831_MOESM6_ESM.pdf]

nature portfolio

Corresponding author(s): Richard M. White

Last updated by author(s): Apr 10, 2023

Reporting Summary

Nature Portfolio wishes to improve the reproducibility of the work that we publish. This form provides structure for consistency and transparency in reporting. For further information on Nature Portfolio policies, see our Editorial Policies and the Editorial Policy Checklist.

Statistics

For all statistical analyses, confirm that the following items are present in the figure legend, table legend, main text, or Methods section.

n/a	Confirmed	
☐	☒	The exact sample size (*n*) for each experimental group/condition, given as a discrete number and unit of measurement
☐	☒	A statement on whether measurements were taken from distinct samples or whether the same sample was measured repeatedly
☐	☒	The statistical test(s) used AND whether they are one- or two-sided<br>*Only common tests should be described solely by name; describe more complex techniques in the Methods section.*
☒	☐	A description of all covariates tested
☐	☒	A description of any assumptions or corrections, such as tests of normality and adjustment for multiple comparisons
☐	☒	A full description of the statistical parameters including central tendency (e.g. means) or other basic estimates (e.g. regression coefficient) AND variation (e.g. standard deviation) or associated estimates of uncertainty (e.g. confidence intervals)
☐	☒	For null hypothesis testing, the test statistic (e.g. *F*, *t*, *r*) with confidence intervals, effect sizes, degrees of freedom and *P* value noted<br>*Give P values as exact values whenever suitable.*
☒	☐	For Bayesian analysis, information on the choice of priors and Markov chain Monte Carlo settings
☒	☐	For hierarchical and complex designs, identification of the appropriate level for tests and full reporting of outcomes
☐	☒	Estimates of effect sizes (e.g. Cohen's *d*, Pearson's *r*), indicating how they were calculated

Our web collection on statistics for biologists contains articles on many of the points above.

Software and code

Policy information about availability of computer code

Data collection	No custom software was used to collect data.
Data analysis	Data analysis was performed through GraphPad Prism 9.1.1, R Studio 4.0.5, MATLAB R2020a, FIJI 2.1.0, Agilent Wave Software and FlowJo 10.8.1. Packages used: CellRanger 6.0.2, Seurat 4.0.2, fgsea 1.16.0, Go.db 3.12.1, FASTQC 0.11.9, TRIMMOMATIC 14.0.1, Salmon 1.4.0, DESeq2 1.30.1. Image segmentation available on Github (https://github.com/dlumaquin/LDmelanoma). Full analysis is outlined in the manuscript.

For manuscripts utilizing custom algorithms or software that are central to the research but not yet described in published literature, software must be made available to editors and reviewers. We strongly encourage code deposition in a community repository (e.g. GitHub). See the Nature Portfolio guidelines for submitting code & software for further information.

Data

Policy information about availability of data

All manuscripts must include a data availability statement. This statement should provide the following information, where applicable:

- Accession codes, unique identifiers, or web links for publicly available datasets
- A description of any restrictions on data availability
- For clinical datasets or third party data, please ensure that the statement adheres to our policy

The sequencing data generated in this study using danio rerio GRCz11 have been deposited in the Gene Expression Omnibus database under accession code

GSE201378 [https://www.ncbi.nlm.nih.gov/geo/query/acc.cgi?acc=GSE201378]. The processed sequencing data are provided as Supplementary Tables. The sequencing data and key findings generated in this study are provided in the Supplementary Information/Source Data file. The single cell RNA-sequencing data from Smalley et al used in this study24 are available in the GEO database under accession code GSE174401 [https://www.ncbi.nlm.nih.gov/geo/query/acc.cgi?acc=GSE174401]. The CCLE and TCGA data used in this study are available in the Depmap database [https://depmap.org/portal/download/all/] and TCGA database [https://tcga.xenahubs.net]. Source data are provided as a Source Data file.

Human research participants

Policy information about studies involving human research participants and Sex and Gender in Research.

Reporting on sex and gender	n/a
Population characteristics	n/a
Recruitment	n/a
Ethics oversight	n/a

Note that full information on the approval of the study protocol must also be provided in the manuscript.

Field-specific reporting

Please select the one below that is the best fit for your research. If you are not sure, read the appropriate sections before making your selection.

☒ Life sciences ☐ Behavioural & social sciences ☐ Ecological, evolutionary & environmental sciences

For a reference copy of the document with all sections, see nature.com/documents/nr-reporting-summary-flat.pdf

Life sciences study design

All studies must disclose on these points even when the disclosure is negative.

Sample size	Experiments were completed with at least n =3 independent biological replicates. For sequencing, at least n = 3 zebrafish were dissected per sequencing reaction to minimize variability.
Data exclusions	No data was excluded from the analysis other than quality control filtering.
Replication	Experiments were independently repeated at least three times with technical replicates as reported in the manuscript. All attempts of replication were successful.
Randomization	Animals were derived from the same tank but randomized for injection and tumor generation. For cell culture experiments, cells were derived from the same passage before seeding in different wells for treatment conditions.
Blinding	Image analysis for lipid droplets was blinded: one investigator acquired images while another investigator quantified lipid droplets in coded file names. Otherwise blinding was not utilized in this study as it was not feasible to perform due to the scope of the genetic and pharmacological experiments performed.

Reporting for specific materials, systems and methods

We require information from authors about some types of materials, experimental systems and methods used in many studies. Here, indicate whether each material, system or method listed is relevant to your study. If you are not sure if a list item applies to your research, read the appropriate section before selecting a response.

Materials & experimental systems

n/a	Involved in the study	
☐	☒	Antibodies
☐	☒	Eukaryotic cell lines
☒	☐	Palaeontology and archaeology
☐	☒	Animals and other organisms
☒	☐	Clinical data
☒	☐	Dual use research of concern

Methods

n/a	Involved in the study	
☒	☐	ChIP-seq
☐	☒	Flow cytometry
☒	☐	MRI-based neuroimaging

Antibodies

Antibodies used	BRAFV600E, Abcam, ab228461<br>PTEN, Cell Signaling, 9559S<br>CD117, Invitrogen, #17-1179-42<br>p75 anti-CD271 (FITC), BioLegend, #345104<br>PLIN2, Proteintech, 15294-1-AP<br>Anti-rabbit AF555, Cell Signaling, 4413S
Validation	BRAFV600E - Antibody has been validated by the manufacturer (https://www.abcam.com/products/primary-antibodies/braf-mutated-v600e-antibody-ve1-ab228461.html) and Histowiz for IHC.<br>PTEN - Antibody has been validated by the manufacturer for IHC (https://www.cellsignal.com/products/primary-antibodies/pten-138g6-rabbit-mab/9559). Data shown for Supplementary Figure 4a.<br>CD117 - Antibody has been validated by the manufacturer for flow cytometry (https://www.thermofisher.com/antibody/product/CD117-c-Kit-Antibody-clone-YB5-B8-Monoclonal/17-1179-42) and used by Baggiolini et al., Science, 2021.<br>p75 - Antibody has been validated by the manufacturer for flow cytometry (https://www.biolegend.com/en-us/productstab/fitc-anti-human-cd271-ngfr-antibody-6209?GroupID=GROUP28) and used by Baggiolini et al., Science, 2021.<br>PLIN2 - Antibody has been validated by the manufacturer for immunofluorescence (https://www.ptglab.com/products/ADRP-Antibody-15294-1-AP.htm) and structures resembling lipid droplets visualized in Figure 3c.<br>Anti-rabbit AF555 - Antibody has been validated by the manufacturer for immunofluorescence (https://www.cellsignal.com/products/secondary-antibodies/anti-rabbit-igg-h-l-f-ab-2-fragment-alexa-fluor-555-conjugate/4413).

Eukaryotic cell lines

Policy information about cell lines and Sex and Gender in Research

Cell line source(s)	Human A375, RPMI-7951, A2058, SKMEL5 and SKMEL28 cells were purchased from ATCC. hPSCs were derived from H9 (WA-06, passage 40-60, WiCell).
Authentication	Human A375, RPMI-7951, A2058, SKMEL5 and SKMEL28 cells were authenticated using Short Tandem repeat profiling at ATCC. hPSC cells were assessed for genomic integrity by karyotyping.
Mycoplasma contamination	All cell lines were confirmed mycoplasma negative using a commercially available mycoplasma kit.
Commonly misidentified lines (See ICLAC register)	No commonly misidentified cell lines were used in this study.

Animals and other research organisms

Policy information about studies involving animals; ARRIVE guidelines recommended for reporting animal research, and Sex and Gender in Research

Laboratory animals	Species: danio rerio (zebrafish)<br>Strain: mitfa-/-, mpv17-/-, mitfa:BRAFV600E, p53-/-<br>Age: 0-4 dpf embryos, 3-6 month adults
Wild animals	No wild animals were used in this study.
Reporting on sex	Matched ratios of male to female fish per genotype were used to generate melanomas using the TEAZ model.
Field-collected samples	No field collected samples were used in this study.
Ethics oversight	All studies comply with institutional ethics regulations. All zebrafish experiments are approved by MSKCC IACUC protocol number 12-05-008.

Note that full information on the approval of the study protocol must also be provided in the manuscript.

Flow Cytometry

Plots

Confirm that:

☒ The axis labels state the marker and fluorochrome used (e.g. CD4-FITC).

☒ The axis scales are clearly visible. Include numbers along axes only for bottom left plot of group (a 'group' is an analysis of identical markers).

☒ All plots are contour plots with outliers or pseudocolor plots.

☒ A numerical value for number of cells or percentage (with statistics) is provided.

Methodology

Sample preparation	Cells were incubated overnight with 100 µM oleic acid then trypsinized and washed with PBS. Cells were stained for viability with 1:1000 DAPI for five minutes, passed through a 40 µm filter and PLIN2-tdTomato signal was assessed via flow cytometry according to Lumaquin et al., Elife, 2021.<br><br>Dox-inducible BRAFV600E PTEN KO hPSC-derived melanoblasts were sorted at day 11 of differentiation according to Baggiolini et al., Science, 2021. The cells in differentiation were initially dissociated into single cells using Accutase (Innovative Cell Technologies, 397) for 20 minutes at 37°C and then stained with a conjugated antibody against cKIT (Anti-Hu CD117 (cKIT) (APC), Invitrogen #17-1179-42) and P75 (anti-CD271 (FITC), BioLegend #345104). Cells double positive for FITC (P75) and APC (cKIT) were sorted and DAPI was used to exclude dead cells.
Instrument	Beckman Coulter CytoFLEX Flow Cytometer and BD FACS Aria6
Software	FlowJo 10.8.1
Cell population abundance	ZMEL-LD cells were not collected post-sort. For hPSC melanoblasts, cells were either expanded as melanoblasts or differentiated into melanocytes according to the manuscript. Cells were imaged showing morphological features of melanoblast spheroids or pigment producing melanocytes prior to seeding for Seahorse experiments.
Gating strategy	FSC-A/SSC-A gates of the starting cell population was used to identify viable cells from debris. Singlet analysis was performed using SSC-A/SSC-H gating. Viable cells were identified using DAPI negative staining. For ZMEL-LD, the GFP positive population was gated. PLIN2-tdTomato positive "lipid droplet positive" was determined using a GFP positive/tdTomato negative population in Supplementary Figure 8b. For hPSC melanoblasts, the cKIT positive/p75 positive population was gated and sorted for expansion or differentiation according to Baggiolini et al., Science, 2021.

☒ Tick this box to confirm that a figure exemplifying the gating strategy is provided in the Supplementary Information.

March 2021
